# Emerging Trends in Streptococcal Toxic Shock Syndrome, Japan

**DOI:** 10.3201/eid3104.241076

**Published:** 2025-04

**Authors:** Mugen Ujiie

**Affiliations:** National Center for Global Health and Medicine, Tokyo, Japan

**Keywords:** *Streptococcus pyogenes*, streptococcal toxic shock syndrome, STSS, streptococcal infections, M1UK lineage, bacteria, streptococci, Japan

## Abstract

Japan experienced substantial increases in streptococcal toxic shock syndrome and group A *Streptococcus* pharyngitis after relaxing COVID-19 restrictions in May 2023. Increased detection of the M1_UK_ lineage of *Streptococcus pyogenes,* especially in the vicinity of Tokyo, emphasizes the need to raise awareness of disease characteristics and epidemiologic trends.

A genetic variant of *Streptococcus pyogenes*, the M1_UK_ lineage, is characterized by high transmissibility and increased production of streptococcal pyrogenic exotoxin A. This variant might have been associated with increased scarlet fever cases in England since ≈2014 (*1*). Similar trends have been observed in other regions, including Europe and North America (*2*,*3*). In New Zealand, M1_UK_ strains have been detected among school children with pharyngitis, suggesting that community transmission might be a source of invasive infections (*4*). We document a similar trend in Japan since ≈2014 (*5*–*7*) and demonstrate increased prevalence of streptococcal toxic shock syndrome (STSS) and group A *Streptococcus* (GAS) pharyngitis (Figure 1). The trend in Japan was temporarily disrupted by nonpharmaceutical interventions during the COVID-19 pandemic but resurged in 2023 after relaxation of COVID-19 restrictions, similar to the case for other infectious diseases. This report might inform effective infection control strategies and raise awareness for STSS. Ethics approval was not required for this registered study, which used routine surveillance data.

**Figure 1:**
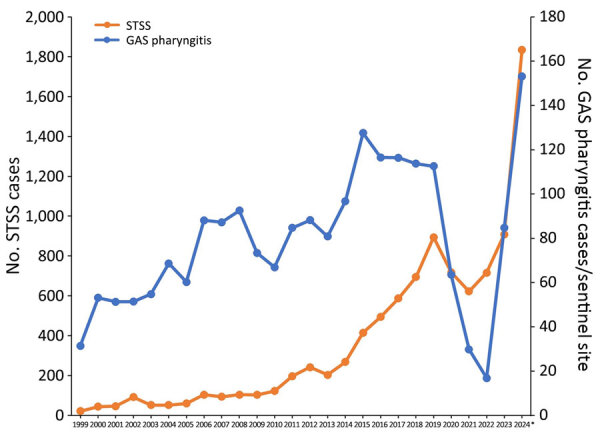
Number of reported STSS (all-cause, including GAS and other streptococcal groups) and GAS pharyngitis reported in Japan during 1999–2024. GAS pharyngitis is reported weekly from ≈3,000 pediatric sentinel medical institutions nationwide. Scales for the y-axes differ substantially to underscore patterns but do not permit direct comparisons. *Data for 2024 through week 50. GAS, group A *Streptococcus*; STSS, streptococcal toxic shock syndrome.

Under the Infectious Diseases Control Law in Japan, physicians must report STSS cases that meet criteria for shock symptoms and >2 of the following conditions: hepatic failure, renal failure, acute respiratory distress syndrome, disseminated intravascular coagulation, soft tissue inflammation, generalized erythematous rash, central nervous system symptoms, or detection of β-hemolytic streptococci from typically sterile sites (e.g., blood). The Japan National Institute of Infectious Diseases reported a marked increase in STSS cases in 2024 (*8*). By week 50 (December 15) of 2024, a total of 1,834 cases had been reported, which is the highest annual number of reported cases (Figure 1). By week 24 (June 16) of 2024, a total of 1,060 STSS cases had been reported; 656 cases were caused by GAS, 222 by group G *Streptococcus*, 114 by group B *Streptococcus*, 10 by group C *Streptococcus*, and 58 by other or unknown groups (*8*). The percentage of STSS cases caused by GAS rose from 30%–50% during 2018–2023 to 62% in 2024. 

By June 19, 2024, the National Institute of Infectious Diseases had received 532 isolates from patients with STSS in 42 prefectures (*8*). GAS accounted for 377 (70.9%) of those cases; 221 (58.6%) were M1 strains, of which 194 (87.8%) belonged to the M1_UK_ lineage (Figure 2). The highest prevalence of M1_UK_ lineage strains was seen in the Kanto region and surrounding areas. The highest number of M1_UK_ isolates were reported in Tokyo (n = 47), followed by Kanagawa (n = 20), Chiba (n = 15), Nagano (n = 9), and Saitama (n = 8). Analysis of 760 GAS isolates from patients with STSS during 2018–2023 identified 215 (28.3%) as M1 strains; 50 (23.3%) of those belonged to the M1_UK_ lineage (Figure 2) (*8**,**9*). Those data indicate a substantial rise in M1_UK_ lineage strains since 2023, especially in Japan’s Kanto region.

**Figure 2:**
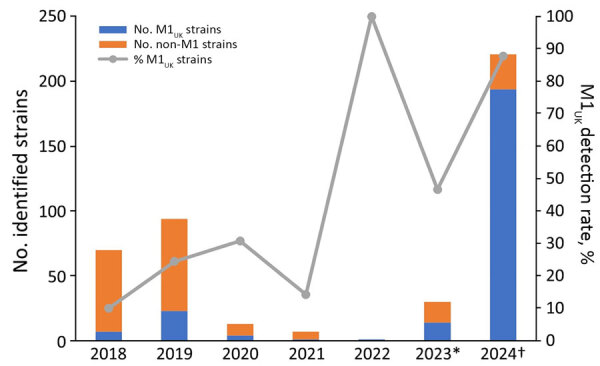
Prevalence of *Streptococcus pyogenes* M1 strains in study of emerging trends in streptococcal toxic shock syndrome, Japan. M1_UK_ lineage within *emm1* genotype strains detected in Japan during 2018–2024 is indicated. Detection rate of the M1_UK_ lineage increased after COVID-19 quarantine measures were lifted in the 19th week of 2023 and was higher in 2024 than at corresponding periods in 2023. *Data for 2023 through November 30. †Data for 2024 data through June 19.

Among the 656 patients with STSS caused by GAS, 377 (57%) were male and 279 (43%) female (*8*). The age distribution was skewed toward the elderly. Age ranges were <20 (n = 23), 20–29 (n = 11), 30–39 (n = 55), 40–49 (n = 87), 50–59 (n = 98), 60–69 (n = 132), 70–79 (n = 140), and >80 (n = 110) years.

Although reporting clinical outcomes is not mandatory, among the 656 STSS cases caused by GAS, 149 deaths (87 male and 62 female patients) occurred at diagnosis (*8*). No deaths occurred in patients <20 years of age; deaths occurred in patients 20–29 (n = 2), 30–39 (n = 12), 40–49 (n = 16), 50–59 (n = 16), 60–69 (n = 34), 70–79 (n = 36), and >80 (n = 33) years of age. Deaths occurred in 30.9% (21/68) of persons <50 years of age during July–December 2023, a marked increase from previous years (19.7% in 2018, 24.1% in 2019, 12.8% in 2020, 9.1% in 2021, 12.1% in 2022, and 15.4% during January–June 2023) (*9*).

The increase in detection and prevalence of M1_UK_ lineage strains in Japan, especially in the Kanto region around Tokyo, and the correlation between the rise in GAS-caused STSS cases and the increase M1_UK_ bacteria isolation rates is concerning. The rising incidence of STSS, characterized by a high mortality rate and requiring prompt treatment for invasive GAS infections, raises critical public health concerns.

In conclusion, the resurgence of STSS and GAS pharyngitis in Japan after relaxation of COVID-19 restrictions highlights the need for continuous surveillance and public health preparedness. The increasing detection of the *S. pyogenes* M1_UK_ lineage emphasizes the importance of genetic monitoring and targeted interventions to prevent its spread. Effective infection control strategies, heightened awareness among healthcare professionals, and public health education are essential to address severe infections in the postpandemic era (*10*).
